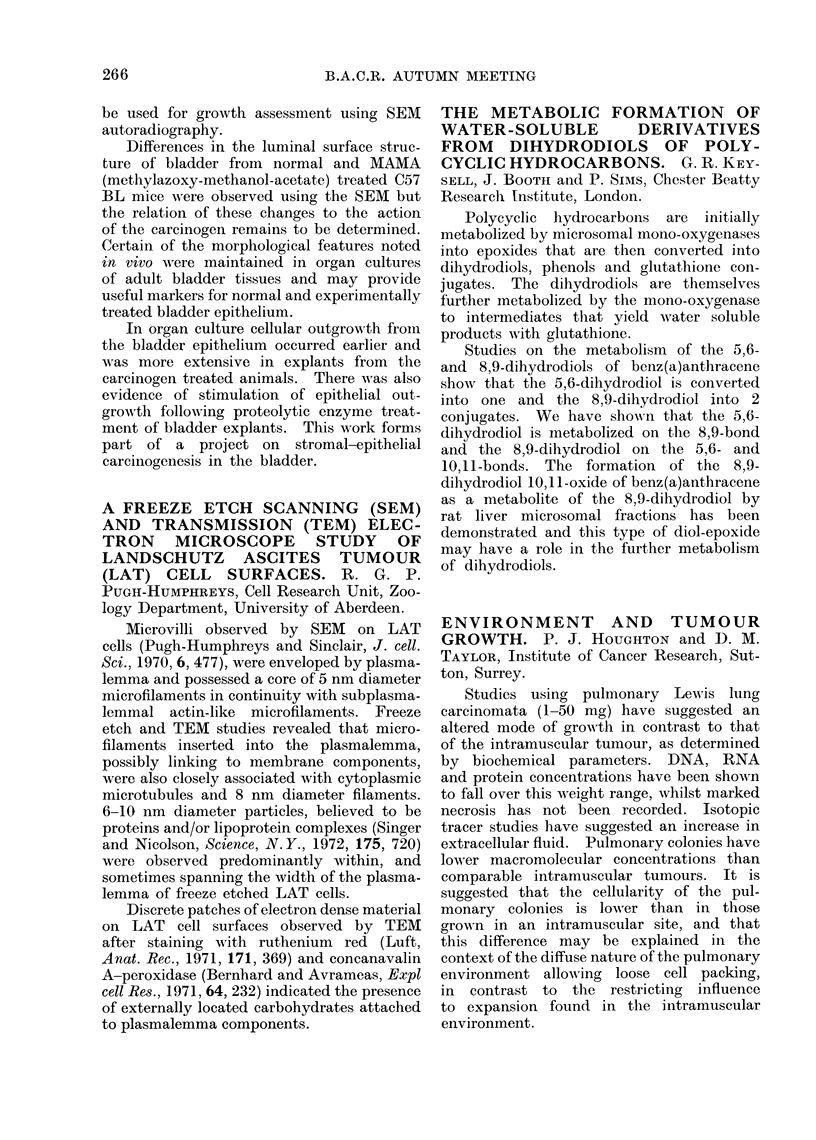# Proceedings: Environment and tumour growth.

**DOI:** 10.1038/bjc.1975.58

**Published:** 1975-02

**Authors:** P. J. Houghton, D. M. Taylor


**ENVIRONMENT AND TUMOUR GROWTH.** P. J. HOUGHTON and D. M. TAYLOR, Institute of Cancer Research, Sutton, Surrey.

Studies using pulmonary Lewis lung carcinomata (1–50 mg) have suggested an altered mode of growth in contrast to that of the intramuscular tumour, as determined by biochemical parameters. DNA, RNA and protein concentrations have been shown to fall over this weight range, whilst marked necrosis has not been recorded. Isotopic tracer studies have suggested an increase in extracellular fluid. Pulmonary colonies have lower macromolecular concentrations than comparable intramuscular tumours. It is suggested that the cellularity of the pulmonary colonies is lower than in those grown in an intramuscular site, and that this difference may be explained in the context of the diffuse nature of the pulmonary environment allowing loose cell packing, in contrast to the restricting influence to expansion found in the intramuscular environment.